# Continuous renal replacement therapy in patients with HIV/AIDS

**DOI:** 10.1186/s12882-020-01754-4

**Published:** 2020-03-11

**Authors:** Hebing Guo, Jingyuan Liu, Lin Pu, Jingjing Hao, Ningning Yin, Yufeng Liu, Haofeng Xiong, Ang Li

**Affiliations:** Department of Critical Care Medicine, Beijing Ditan Hospital, Capital Medical University, No. 8 Jingshundong Street, Beijing, 100015 Chaoyang District China

**Keywords:** HIV/AIDS, CRRT, Mortality, Risk factors

## Abstract

**Background:**

Acute kidney injury (AKI) is a common complication among human immunodeficiency virus (HIV)-infected patients resulting in increased morbidity and mortality. Continuous renal replacement therapy (CRRT) is a useful method and instrument in critically ill patients with fluid overload and metabolic disarray, especially in those who are unable to tolerate the intermittent hemodialysis. However, the epidemiology, influence factors of CRRT and mortality in patients with HIV/AIDS are still unclear in China. This study aims to study the HIV-infected patients admitted in Intensive Care Unit (ICU) and explore the influence factors correlated with CRRT and their prognosis.

**Methods:**

We performed a retrospective case-control study in the ICU of the Beijing Ditan Hospital Capital Medical University. From June 1, 2005 to May 31, 2017, 225 cases were enrolled in this clinical study.

**Results:**

122 (54.2%) patients were diagnosed with AKI during their stay in ICU, the number and percentage of AKI stage 1, 2 and 3 were 38 (31.1%), 21(17.2%) and 63(51.7%), respectively. 26.2% of AKI patients received CRRT during the stay of ICU. 56.25% CRRT patients died in ICU. The 28-day mortality was 62.5%, and the 90-day mortality was 75%. By univariate logistics analysis, it showed that higher likelihood of diagnosis for respiratory failure (OR = 7.333,95% CI 1.467–36.664, *p* = 0.015), higher likelihood of diagnosis for septic shock (OR = 1.005,95% CI 1.001–1.01, *p* = 0.018), and higher likelihood to use vasoactive agents (OR = 10.667,95% CI 1.743–65.271, *p* = 0.001), longer mechanical ventilation duration (OR = 1.011,95% CI 1.002–1.019, *p* = 0.011), higher likelihood for diagnosis for PCP (OR = 7.50,95% CI 1.288–43.687, *p* = 0.025), higher SOFA score at ICU admission (OR = 1.183,95% CI 1.012–1.383, *p* = 0.035), longer duration of CRRT (OR = 1.014,95% CI 1.001–1.028, *p* = 0.034) contributed to a higher mortality at ICU. The Cox Analysis for the cumulative survival of AKI 3 patients between the CRRT and non-CRRT groups shows no significant differences (*p* = 0.595).

**Conclusions:**

There is a high incidence of AKI in HIV-infected patients admitted in our ICU. Patients with severe AKI were more prone to be admitted for CRRT and have a consequent poor prognosis.

## Introduction

Acute kidney injury (AKI) is a common disease in critical patients and affects more than 13 million people all around the world annually [1]. Patients who are infected by human immunodeficiency virus (HIV) are more vulnerable to suffer from AKI and showing a higher mortality and morbidity [2, 3]. 66% HIV-infected patients are diagnosed with AKI during their stay in the Intensive Care Unit (ICU) [4]. Continuous renal replacement therapy (CRRT) has been an important maintenance intervention in patients with severe AKI for more than one decade [5, 6]. However, few studies have clarified CRRT in patients with HIV/AIDS. Epidemiology and influence factors of CRRT in patients with HIV/AIDS are still unclear in China. This study aims to make these determinations among HIV-infected individuals in an observational retrospective clinical study.

## Methods

### Study design and population

The study is a retrospective research in ICU of the Beijing Ditan Hospital Capital Medical University which is one of the top three hospital for infectious diseases. About five hundred patients were admitted in this general ICU and there were 20-bed ward for both medical and surgical patients each year. We reviewed the Hospital Information management System (HIS) from June 1, 2005 to May 31, 2017 in Beijing Ditan Hospital Capital Medical University for this study. All of the databases were recorded and analyzed by infectiology specialists from our team. Patients who were clearly diagnosed with HIV and older than18 years old were included in this study. Patients who underwent RRT before admission, stayed in ICU less than 48 h and were admitted in ICU more than once within 6 months, were excluded from this research. This study only recorded the first admission for patients who were admitted at ICU more than once within 6 months.

There were 284 cases admitted at ICU of Beijing Ditan Hospital Capital Medical University from June 1, 2005 to May 31, 2017. Forty seven cases were excluded because of the length of stay at ICU less than 48 h. Four cases were excluded by accepting renal replacement therapy before admission. Meanwhile 8 patients were recorded the first admission because of admitting at ICU twice within 6 months. A total of 225 cases were considered for this study.

No trial of any specific therapeutic or prophylactic intervention was carried out and only observational data were used for our analysis. This study was approved by the Ethics Committee of Beijing Ditan Hospital, Capital Medical University, No. 2018–005-01.

### Definitions

#### AKI diagnosis

We used the Kidney Disease Improving Global Outcomes (KDIGO) criteria for the diagnosis of AKI. As defined by the KDIGO, AKI would be diagnosed if there is a 50% rise in serum creatinine from baseline at any point during the patient’s prior 7-day ICU admission; a rise in serum creatinine of > 26.5 mmol/L within a 48-h period; a fall in urine output of < 0.5 mL/kg/h for more than 6 h; an absolute serum creatinine of > 353.6 mmol/L; or an RRT is initiated [7].

#### HIV diagnosis

As defined by the US Centers for Disease Control and Prevention (CDC) in a revised surveillance case definition for HIV infection in 2014, HIV infection is diagnosed when a positive result from HIV antibody or combination of an antigen/antibody test is obtained, and another positive result is obtained from a supplemental different HIV test, performed in parallel or subsequently to the initial test [8]. Alternatively, HIV infection is diagnosed if a positive result or report of a detectable quantity from any of the following HIV virologic tests has been obtained:

- Qualitative HIV NAT (DNA or RNA)– Quantitative HIV NAT (viral load assay)
- HIV-1 p24 antigen test
- HIV isolation (viral culture) or
- HIV nucleotide sequence (genotype) [9]

### Data collection

The following demographic, clinical, laboratory data, complication and multiple organs support therapy were collected from the patient medical manuals and an electronic hospital database by four investigators (H. Guo, L. Pu, J. Hao, and N. Yin): age, sex, body mass index, lymphocyte count, hemoglobin, albumin, t-bil, serum calcium, lactate, sodium bicarbonate, serum phosphate, Glomerular Filtration Rate (GRF), Mean Platelet Volume (MPV), Alanine Transaminase (ALT), glutamic oxalacetic transaminase (AST), Lactate Dehydrogenase (LDH),CD4 count at ICU admission, lowest CD4 count in ICU, Highly Active Anti-Retroviral Therapy (HAART), Pneumocystis Carinii Pneumonia (PCP), septic shock, respiratory failure, the use of vasoactive agents, duration of mechanical ventilation, duration of CRRT.

The severity of illness was evaluated by the Sequential Organ Failure Assessment (SOFA) and the Acute Physiology and Chronic Health Evaluation (APACHE)II score, which calculation was based on the worst variables recorded within the first 24 h of ICU admission.

All patients received CRRT through continuous venovenous hemodiafiltration (CVVHDF) (therapeutic dose of 30 ml/kg) and the related data were not recorded in this study.

Length of ICU stay and hospital were recorded in the Electronic Hospital Database.

### Outcome measures

Outcome was recorded by the HIS of Beijing Ditan Hospital Capital Medical University. Follow-up visit was conducted by infectious diseases specialists by telephone in this study.

### Statistical analysis

Categorical variables were compared using a chi-squared test for trends. Risk factors were assessed by univariate analyses, and variables that were statistically significant (*p* < 0.05) in the univariate analyses were later included in a multivariate analysis. Univariate and multivariate analyses were performed using a logistic regression. The life table method was used to determine survival curves, and a Cox proportional hazards model was used to evaluate the statistical differences between the survival and non-survival curves. Data are presented as mean ± standard deviation, median (interquartile range), percentage of number of cases, odds ratios (ORs) with 95% confidence intervals. A two-tailed *p* < 0.05 was considered significant. Statistical analyses were performed using the SPSS version 22.0 software (SPSS, Inc., Chicago, IL).

## Results

### Patients characteristics

**Fig. 1:**
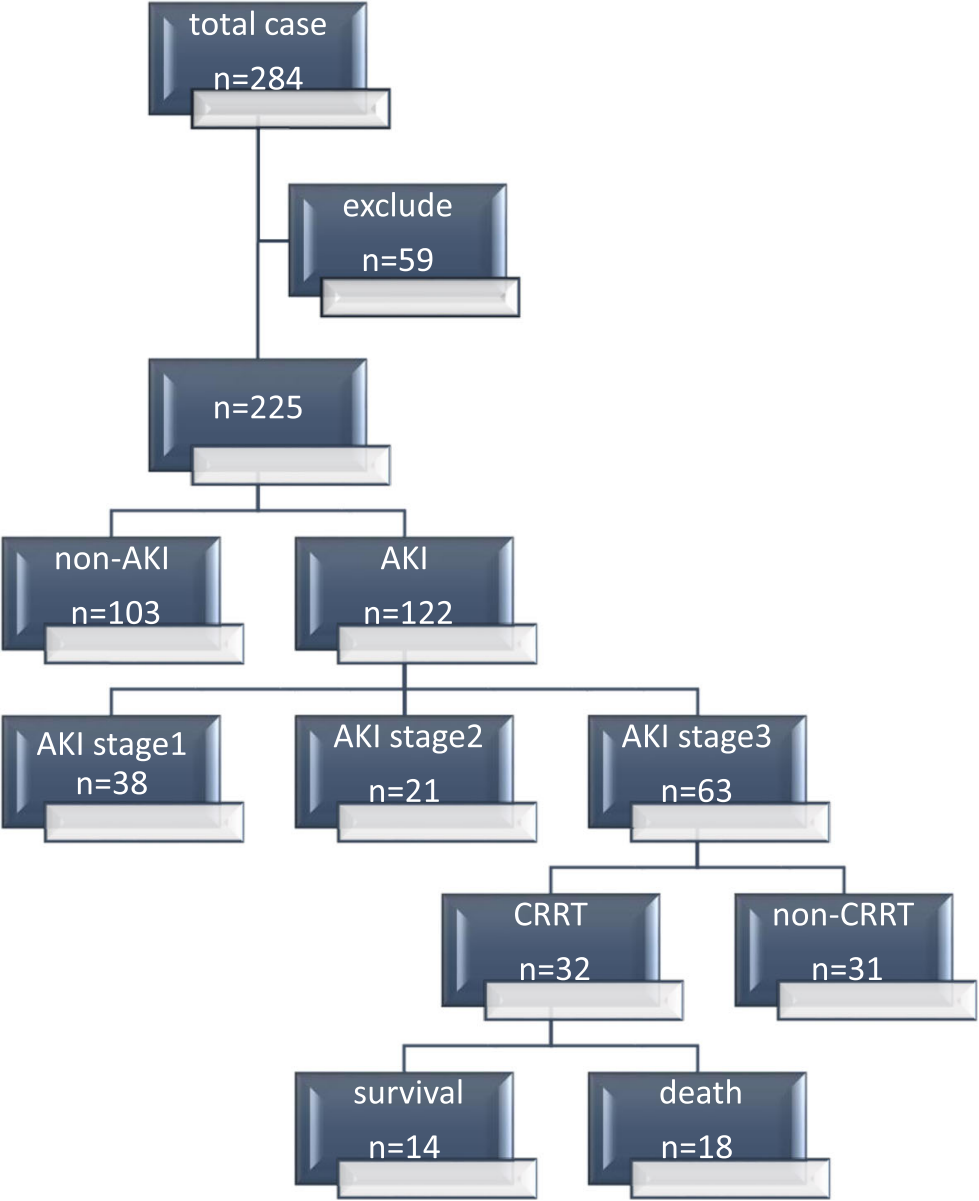
Study flow chart detailing the inclusion and exclusion of patients

Among the 225 patients, 205 (91.1%) were male. The average age of the total numbers was 42 ± 13 years. 122(54.2%) patients were diagnosed with AKI during their stay at ICU. As shown in Fig. 1, the number and percentage of AKI stage 1/2/3 were respectively 38 (31.1%)/21 (17.2%)/63 (51.7%). Thirty two patients (about 26.2% of total AKI) received continuous renal replacement therapy (CRRT) at ICU.56.25% (*n* = 18) of patients who received CRRT died at ICU.

Comparison between AKI patients with CRRT and without are shown in Table 1. Hemoglobin and CD4 counts at ICU admission had a statistically significant difference (*p* = 0.048 and *p* = 0.043, respectively). As per complications, the incidence of *Pneumocystis carinii* pneumonia (PCP) and respiratory failure showed a statistical significant difference (*p* = 0.006 and *p* = 0.01, respectively). In severity of illness, patients with CRRT were even worse both in SOFA and APACHE-II score (*p*<0.001, and *p*<0.001, respectively). Patients with CRRT stayed in the hospital for a shorter period than those without (*p* = 0.022).

**Table 1:** Demographic and Clinical Characteristics of 122 AKI Patients Admitted to the Intensive Care Unit

	Patients with AKI *n* = 122	Patients with CRRT *n* = 32	Patients without CRRT *n* = 90	*p*
Male(%)	112 (91.8)	31 (96.9)	81 (90)	0.452
Age(years)	41 (34–50)	42 (37–55.5)	41 (31–50)	0.137
**Blood routine and biochemical items**				
Lymphocyte count(× 10^9^)	0.5 (0.3–0.92)	0.54 (0.34–1.10)	0.5 (0.30–0.80)	0.205
Hemoglobin(g/L)	98.9 ± 27.4	90.7 ± 23.8	101.8 ± 28.1	**0.048**
Albumin (g/L)	28.0 ± 5.8	29.3 ± 4.9	27.5 ± 6.1	0.154
Total Bilirubin (u mol/L)	8.65 (5.38–13.43)	7.75 (5.03–15.33)	9.15 (5.4–13.43)	0.682
Serum calcium(mmol/L)	1.92 (1.77–2.09)	2.00 (1.67–2.19)	1.92 (1.82–2.05)	0.375
**Items related to HIV infection**				
CD4 counts at ICU admission	23 (8–78)	37 (13–122)	15 (7–69)	**0.043**
Patients with HAART	30 (24.6)	11 (34.4)	19 (21.1)	0.135
**Items related to AKI**				
The first Creatinine in Hospital (u mol/L) 72.7 (54–129.3)		220.1 (70.9–499.1)	64.5 (51.8–92.6)	**<0.001**
The highest Creatinine before in ICU(u mol/L)48.9 (34.7–70.8)		194.2 (55.4–441.5)	42.1 (32.8–58.6)	**<0.001**
The highest Creatinine during ICU(u mol/L) 159.6 (85.6–331)		375.4 (293.3–580.0)	121.6 (79.4–210.5)	**<0.001**
**Complication**				
Sepsis (%)	104 (85.2)	25 (78.1)	79 (87.8)	0.245
Septic Shock(%)	74 (60.7)	19 (59.4)	55 (61.1)	0.863
PCP (%)	71 (58.2)	12 (37.5)	59 (65.6)	**0.006**
Respiratory Failure (%)	81 (66.4)	15 (46.9)	65 (72.2)	**0.01**
**Multiple Organs Support Therapy**				
The use of vasoactive agents (%)	81 (66.4)	22 (68.8)	59 (65.6)	0.742
Duration of Mechanical ventilation (h)	131.5 (16–264)	141.5 (5–225.5)	124.5 (24–264.0)	0.788
**Severity of illness**				
SOFA score at ICU admission	8 (4–12)	12 (6–16)	7 (4–10)	**<0.001**
APACHE-II score at ICU admission	20 (15–28)	28 (21–34)	18 (15–23)	**<0.001**
**Outcome**				
Death during in ICU	61 (50)	18 (56.3)	43 (47.8)	0.41
Length of ICU stay (days), median (IQR)	9 (5–16)	8 (5–16)	9 (5–17)	0.616
Length of hospital stay (days), median (IQR)	17 (9–30)	15 (7–21)	20 (10–34)	**0.022**

*ICU* Intensive care unit, *HIV* Human immunodeficiency virus, *HAART* Highly Active Anti-Retroviral Therapy, *PCP* Pneumocystis carinii pneumonia, *CRRT* Continuous renal replacement therapy, *SOFA*, *IQR* Interquartile range, *APACHE-II* Acute physiology and chronic health evaluation, version II, *SOFA* Sequential organ failure assessment, *GFR* Glomerular Filtration Rate, *MPV* Mean Platelet Volume

*p* Value < 0.05 was considered significant and is highlighted in bold

**Fig. 2:**
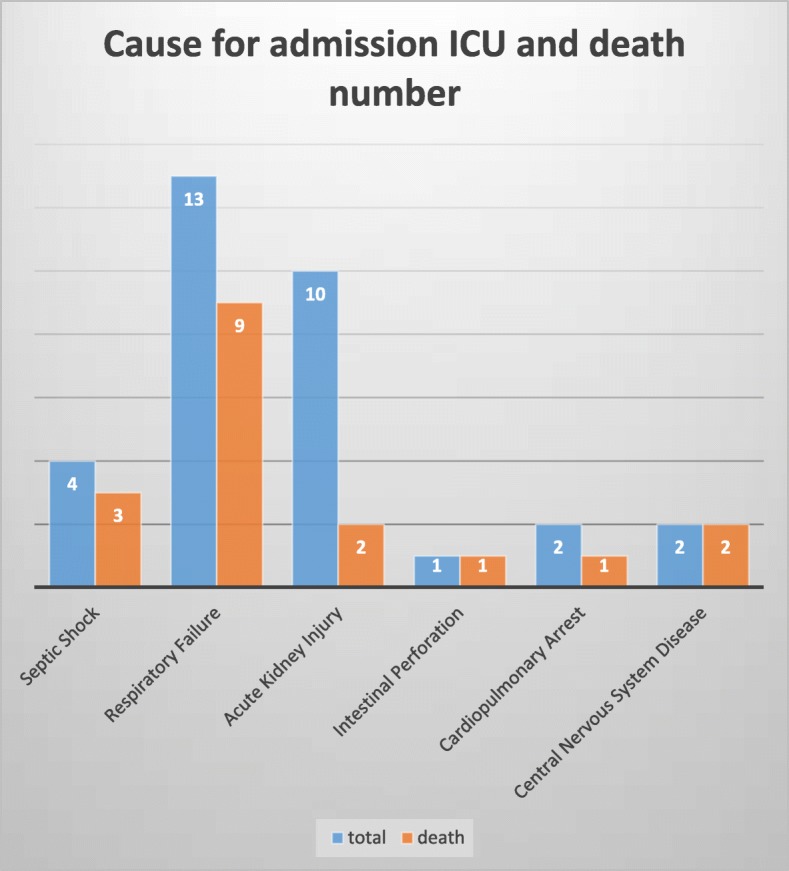
Cause for admission ICU and death number of the 32 CRRT patients

As shown in Fig. 2, 9/13 patients (40.6%) who were admitted at ICU for respiratory failure, died at ICU. Secondly, 2/10 patients (31.25%) who received treatment at ICU mainly for AKI, died at ICU. Septic shock, intestinal perforation, cardiopulmonary arrest, central nervous system disease were the other factors for admission at ICU.

Table 2 shows the strength of association between death at ICU and its potential risk factors, was calculated by univariate logistics analysis. The result showed that respiratory failure, septic shock, the use of vasoactive agents, duration of mechanical ventilation, diagnosis of PCP, SOFA score at ICU admission were independent risk factors for CRRT by univariate logistics analysis.

**Table 2:** Univariate Analysis for the Identification of Predictors the Death of CRRT in ICU

	Odds ratio	95% Confidence interval	*p*
Respiratory Failure	7.333	1.467–36.664	**0.015**
Septic Shock	1.005	1.001–1.010	**0.018**
The use of vasoactive agents	10.667	1.743–65.271	**0.001**
Duration of Mechanical Ventilation	1.011	1.002–1.019	**0.011**
PCP	7.50	1.288–43.687	**0.025**
SOFA score at ICU admission	1.183	1.012–1.383	**0.035**
Duration of CRRT	1.014	1.001–1.028	**0.034**

*CRRT* Continuous renal replacement therapy, *ICU* Intensive care medicine, *SOFA* Sequential organ failure assessment, *PCP* Pneumocystis carinii pneumonia

*p* Value < 0.05 was considered significant and is highlighted in bold

Higher likelihood of diagnosis for respiratory failure (OR = 7.333,95% CI 1.467–36.664, *p* = 0.015), higher likelihood of diagnosis for septic shock (OR = 1.005,95% CI 1.001–1.01, *p* = 0.018), and higher likelihood to use vasoactive agents (OR = 10.667,95% CI 1.743–65.271, *p* = 0.001), longer mechanical ventilation duration (OR = 1.011,95% CI 1.002–1.019, *p* = 0.011), higher likelihood for diagnosis for PCP (OR = 7.50,95% CI 1.288–43.687, *p* = 0.025), higher SOFA score at ICU admission (OR = 1.183,95% CI 1.012–1.383, *p* = 0.035), longer duration of CRRT (OR = 1.014,95% CI 1.001–1.028, *p* = 0.034) contributed to a higher mortality at ICU.

As shown in Fig. 1, Among the AKI stage 3 patients, about a half received CRRT during their stay at ICU. Then we compared the two groups shown in Table 3. Plasma-albumin and CD4 counts at ICU admission had a statistically significant difference (*p* = 0.027 and *p* = 0.009, respectively). In severity of illness, patients who received CRRT had a higher APACHE-II scores (*p* = 0.015). Even with CRRT, the main outcome, such as the mortality at ICU, and the renal recovery in the two groups did not show a significant difference (*p* = 0.521 and *p* = 0.353, respectively). Patients who received CRRT probably had a longer stay tendency at ICU (*p* = 0.679), but a shorter hospital stay (*p* = 0.029).

**Table 3:** The comparison between Patients with CRRT and non-CRRT who were in AKI stage 3

	AKI stage 3 (*N* = 63)	CRRT (*N* = 32)	non-CRRT (*N* = 31)	*p*
Male(%)	57 (90.5)	31 (96.9)	26 (83.9)	0.104
Age(years)	43 (37–50)	42 (37–56)	44 (36–48)	0.527
**Blood routine and biochemical items**				
Lymphocyte count(×10^9^)	0.5 (0.24–1.00)	0.54 (0.34–1.05)	0.5 (0.2–0.8)	0.146
Hemoglobin(g/L)	91 (70.8–108)	91 (71.8–109.5)	89 (70.7–104.5)	0.929
Albumin(g/L)	27.4 (23.8–31.7)	30.3 (24.9–32.5)	26.8 (22.3–30.2)	**0.027**
T-Bil (umol/L)	8.7 (5.8–15.9)	7.8 (5.1–14.5)	11.4 (6.3–16.0)	0.343
Serum calcium(mmol/L)	1.92 (1.71–2.09)	2.0 (1.68–2.18)	1.88 (1.74–2.03)	0.221
Lactate (mmol/L)	2.8 (1.45–6.05)	3.65 (1.55–7.15)	2.1 (1.35–5.0)	0.091
Serum phosphate(mmol/L)	1.11 (0.81–1.65)	1.45 (0.81–1.88)	1.02 (0.81–1.36)	0.159
LDH (U/L)	450 (309.6–786.6)	501 (281.6–1233)	450 (321.2–663.2)	0.336
**Items related to HIV infection**				
CD4 counts at ICU admission(cells/ml)	24 (8–86)	37 (13–116)	11 (5–36)	**0.009**
HIV viral load (copies/mL)	1700 (90–249,112)	1650 (0–174,710)	37,444 (1142–335,614)	0.116
HAART(%)	26 (41)	10 (31)	16 (51.6)	0.052
**Complication**				
PCP (%)	30 (47.6)	12 (37.5)	18 (58.1)	0.102
Septic Shock(%)	41 (65.1)	19 (59.4)	22 (71)	0.245
Multiple Organs Support Therapy				
The use of vasoactive agents(%)	46 (73)	22 (66.8)	24 (77.4)	0.349
Duration of Mechanical ventilation (h)	120 (7–240)	142 (5–256)	119 (14–212.5)	0.825
Severity of illness				
SOFA score at ICU admission (scores)	9 (7–13)	11 (7–14)	9 (6–11)	0.094
APACHE-II score at ICU admission(scores)	23 (19–33)	28 (22–35)	19 (18–30)	**0.015**
**Outcome**				
Death during in ICU	34 (54)	18 (56)	14 (45.2)	0.521
Renal recovery	17 (27.0)	7 (21.9)	10 (32.3)	0.353
Length of ICU stay (days), median (IQR)	8 (5–14)	8 (5–15)	6 (5–14)	0.679
Length of hospital (days), median (IQR)	16 (9–29)	15 (7–21)	17 (12–38)	**0.029**

*T-Bil* Total bilirubin, *HAART* Highly Active Anti-Retroviral Therapy, *PCP* Pneumocystis carinii pneumonia, *CRRT* Continuous renal replacement therapy, *ICU* Intensive care unit, *IQR* Interquartile range, *APACHE-II* Acute physiology and chronic health evaluation, version II, *SOFA* Sequential organ failure assessment

*p* Value < 0.05 was considered significant and is highlighted in bold

**Fig. 3:**
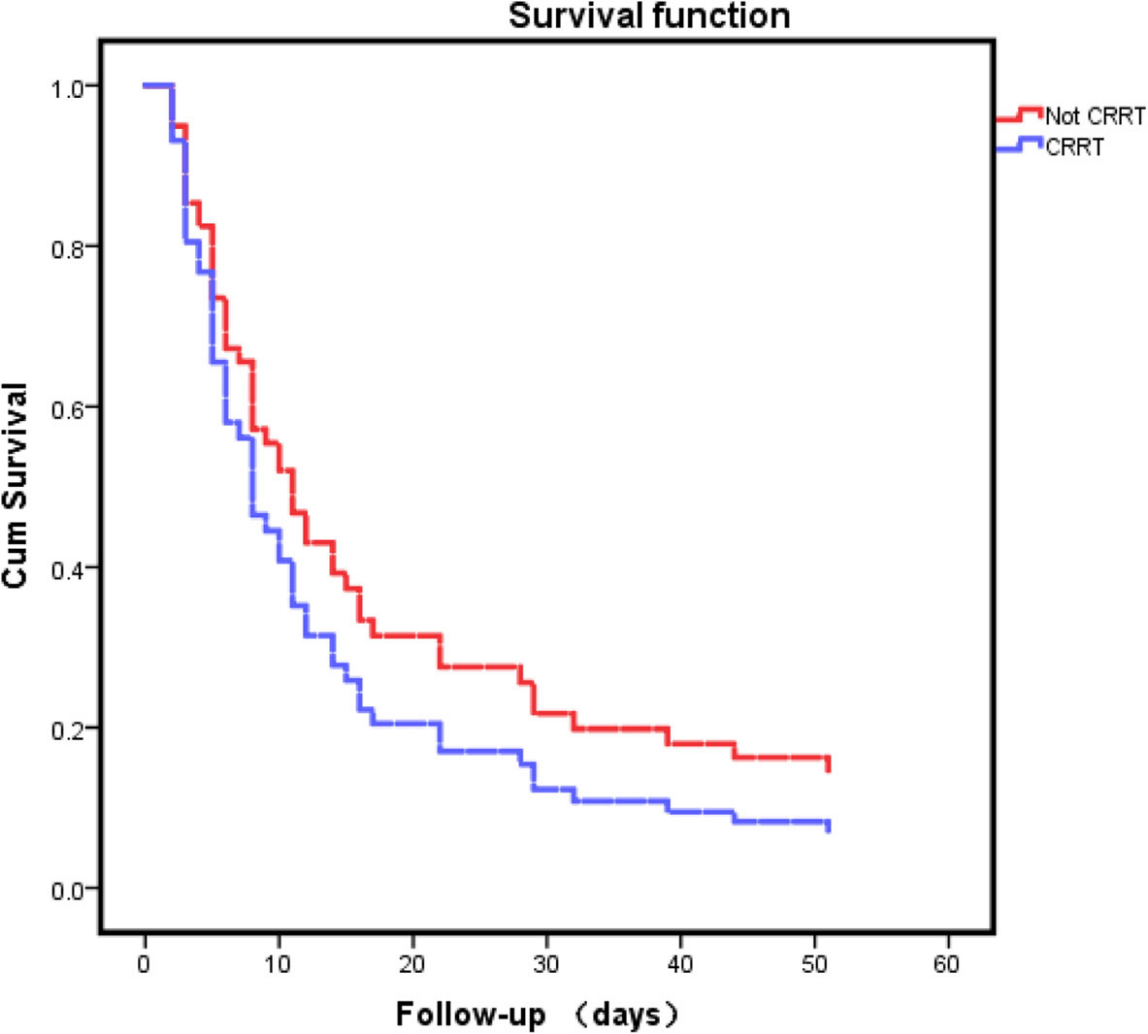
Cumulative survival curves of HIV infected patients who were diagnosed with AKI 3 admitted to the intensive care unit, received CRRT or not received (two groups)

As shown by the cumulative survival curves of HIV-infected patients who were diagnosed with AKI 3 admitted to the intensive care unit (Fig. 3), the group of CRRT and non-CRRT had no significant difference (*p* = 0.595). As determined by the Cox Analysis of the cumulative survival of 3 AKI patients between the CRRT and non-CRRT groups (Table 4), the possibility of using vasoactive agents shows a significant difference on AKI stage 3 patients’ survival (*p* = 0.015, OR = 12.621,95% CI 1.637–97.293).

**Table 4:** The Cox Analysis for the cumulative survival of AKI 3 patients between the CRRT and non-CRRT groups

	Odds ratio	95% Confidence interval	*p*
CRRT	1.236	0.566–2.70	0.595
The possibility of using vasoactive agents	12.621	1.637–97.293	**0.015**
SOFA score at ICU admission	0.978	0.896–1.068	0.625
PCP	0.831	0.362–1.909	0.663

*CRRT* Continuous renal replacement therapy, *ICU* Intensive care unit, *SOFA* Sequential organ failure assessment, *PCP* Pneumocystis carinii pneumonia

*p* Value < 0.05 was considered significant and is highlighted in bold

## Discussion

Lin Pu has concluded that AKI occurred more than 50% on HIV-infected patients admitted to the ICU [10]. Consistent with previous reports: the incidence of AKI for the patients admitted at ICU is 67% [10].CRRT has practically and efficiently used in patients with AKI as a basic equipment [5, 11]. But the occurrence rate and influencing factors to predict patients’ death of CRRT in ICU among the Chinese population is still unclear.

26.2% patients with AKI received CRRT during their stay at ICU in this study, at a significantly higher proportion than 5–6% patients without HIV [2]. The result indicated that HIV-infected patients were more prone to develop severe AKI. Patients with HIV lost kidney function faster than the overall population [12, 13]. Exposure to nephrotoxic drugs such as tenofovir and HIV replicating in other cells besides CD4 cells may aggravate deterioration of the renal function [14–16]. Rasch et al. found that the possibility of HIV patients receiving RRT were more than 4-fold compared with the overall population without RRT. Age, hypertension and AIDS were associated with an increased risk of RRT [14].

There is no difference with a previous study: the mortality ranging from 37 to 88% for the patients admitted at ICU who needed the RRT [17]. The study indicated that 56.25% of CRRT patients died at ICU. Mortality was higher in patients with HIV/AIDS, most likely due to the serious immunosuppression and opportunistic diseases [18]. Compared with survivals with CRRT, 15 non-survived patients suffered from septic shock during their stay at ICU.

Death of patients at ICU was more frequent of they were diagnosed with septic shock, PCP, or had a longer duration of CRRT. Ling ping et al. reported that sepsis-related AKI and using vasopressors were independent risk factors to increase patients’ mortality [19]. Dopamine and norepinephrine are two types of common vasopressors widely used in curing different types of shocks. Patients with septic shock are usually affected by hypotension and coagulopathy [20]. Multiple organ syndrome occurred as a result of ischemia reperfusion injury. Use of vasoactive agents using might increase the risk of ICU death by the poor cardiovascular response to the catecholamine. Chou et al. reported that critically ill patients being treated with a high dose of vasopressor also had a higher ICU mortality, with CRRT not improving patients’ prognosis [21].

Patients with HIV infection are more vulnerable to be affected by opportunistic infections. PCP has been a common manifestation during HIV infection in developed countries. It played an important role throughout the course of the disease and often as the initial manifestation of acquired immunodeficiency syndrome (AIDS) [22]. Meanwhile it resulted in lung injury and gas exchange by inducing a pulmonary inflammatory response. Tumor necrosis factor-α and other immunomodulatory molecules were released as a response to the infection [23, 24]. Prasad et al. found that patients with a higher FiO2 requirements while ventilated and a higher vasoactive agent administration were inclined to have an early (within 24 h) mortality [25]. PCP prophylaxis has been widely used in developed countries [22], but still not commonly used in developing countries. No patients received PCP prophylaxis in this study. On the other hand, 25–33% patients were not diagnosed of PCP before they were clearly diagnosed of HIV [26]. Early diagnosis of HIV-infection and PCP prophylaxis perhaps contributed to a decreased mortality of CRRT.

This study suggests that a longer duration of CRRT can contribute to patients’ death at ICU. Longer duration of CRRT can increase the likelihood of a catheter related bloodstream infection (CRSBI) and more vulnerability of CRRT related complications. Scott found that there was no difference of pediatric survival rate between long and short duration of CRRT [27]. The longer duration of CRRT, the higher risk of complications the patients might have. The patients enrolled in this study were all immunodeficient, therefore any opportunistic infection can be fatal for them.

The 28-day mortality of patients with CRRT was 62.5% in this study, matching with the 62% found in sepsis-induced acute kidney injury patients undergoing CRRT in previous reports [28].

As shown in Fig. 2, 13 patients with a respiratory failure represent the highest proportion of the ICU admission, 10 (76.9%) patients died at ICU. Ten patients of AKI represent the second group of ICU admission, only 2 (20%) died at ICU. Three patients accepted the treatment at ICU because of septic shock, 3 of total (75%) did not survive at ICU. The figure indicated that patients who were initially diagnosed with AKI had probably a better prognosis. On the contrary, the patients who were initially admitted at ICU for respiratory failure or septic shock, usually had a poor outcome compared to patients diagnosed with AKI and needed the help of CRRT.

With the extensively use of highly active antiretroviral therapy (HAART), the survival of HIV-infected patients have been significantly improved [29]. The use of antiretroviral drugs is another determinant factor to compromise the renal function and increase the risk for CRRT [30]. Previous studies indicated that both HIV infection and HAART were directly nephrotoxic [31–34]. We also confirmed that only 30 (24.6%) patients with AKI received the HAART during their stay at ICU, there was no significant difference between the CRRT group and the non-CRRT group. The reason why most patients never received the HAART was that 71.1% patients were diagnosed with HIV infection within 3 months, so the treatment was not started yet [35].

The count of CD4+ cells reflects the immune status of HIV-infected patients. A previous study has proven that CD4 count < 200 cells/ml was an independent predictor of experiencing AKI [36]. Low CD4 count and AIDS are risk factors for HIV-infected people suffering from AKI [37]. Lower CD4 count made HIV patients more vulnerable to suffer from opportunistic infections and increased the risk of kidney injury [35]. The average CD4 count of AKI patients was 23 (8–78) cells/ml, and there was a statistically significant difference (*p* = 0.043) between the CRRT group 37 (13–122) cells/ml and the non-CRRT group 15(7–69) cells/ml. But there was no significant difference (*p* = 0.464) between the CRRT survival group 97 (25–156) cells/ml and the CRRT non-survival group 40 (15–110) cells/ml. CD4 count < 200 cells/ml can lead to immune depression, however, there was no significant difference (*p* = 0.245 and *p* = 0.863) between the CRRT group and the non-CRRT group for sepsis and septic shock. The reason why there was no significant difference between the two groups on CD4 count is probably because the patients admitted at ICU were both suffering for severe immunosuppression and easier to be infected by different pathogens. Franceschini et al. concluded that with the decreasing of patients’ CD4 count, the possibility of morbidity and mortality extensively increased [36].

By using the Cox Analysis for the cumulative survival of AKI 3 patients between the CRRT and non-CRRT groups in Fig. 3, we conclude that CRRT did not improve patients’ long-term survival (*p* = 0.595). Most likely, CRRT had a determinant effect in maintaining the stability of the internal environment and decreasing pre load of heart. While CRRT was a maintenance intervention, other critical conditions except renal injury might affect the outcome. But as the cumulative survival curves illustrated in Fig. 3, APACHE-II score at ICU admission of CRRT and non-CRRT group were separated 28(22–35) and 19(18–30), *p* = 0.015. Data in the Fig. 3 indicated that patients in the CRRT group were more critical. The cumulative survival rate of the CRRT group was lower than the one in the non-CRRT group. The result indicated that AKI 3 might be not the proper stage to start an active intervention. Patients might benefit from an earlier intervention at AKI stage 1 or AKI stage 2.

### Limitations

The number of CRRT events is relatively small, which limits statistical power of analyses. This is a retrospective study. Thus, a large, prospective, randomized, controlled study is needed. Further investigations on different modes and therapeutic dose should be conducted.

## Conclusions

There is a high incidence of AKI in HIV-infected patients admitted in our ICU. Patients with severe AKI were more prone to be admitted for CRRT and have a consequent poor prognosis.

## Data Availability

The data generated and analyzed during the current study are available from the corresponding author on reasonable request.

## Abbreviations

AKI: Acute kidney injury
APACHE-II: Acute physiology and chronic health evaluation, version II
CRRT: Continuous renal replacement therapy
GFR: Glomerular Filtration Rate
HAART: Highly Active Anti-Retroviral Therapy
HIV: Human immunodeficiency virus
ICU: Intensive care unit
IQR: Interquartile range
MPV: Mean Platelet Volume
PCP: Pneumocystis carinii pneumonia
SOFA: Sequential organ failure assessment
T-Bil: Total bilirubin
